# Gut Microbiota Is a Potential Biomarker in Inflammatory Bowel Disease

**DOI:** 10.3389/fnut.2021.818902

**Published:** 2022-01-21

**Authors:** Xue Guo, Chen Huang, Jing Xu, Haoming Xu, Le Liu, Hailan Zhao, Jiaqi Wang, Wenqi Huang, Wu Peng, Ye Chen, Yuqiang Nie, Yongjian Zhou, Youlian Zhou

**Affiliations:** ^1^Department of Gastroenterology and Hepatology, The Second Affiliated Hospital, School of Medicine, South China University of Technology, Guangzhou, China; ^2^Department of Gastroenterology and Hepatology, Guangzhou First People's Hospital, School of Medicine, South China University of Technology, Guangzhou, China; ^3^Department of Gastroenterology, Shenzhen Hospital, Southern Medical University, Shenzhen, China

**Keywords:** inflammatory bowel disease, ulcerative colitis, Crohn's disease, biomarkers, gut microbiome, fecal microbiota transplantation

## Abstract

Inflammatory bowel disease (IBD), which includes ulcerative colitis (UC) and Crohn's disease (CD), is characterized by relapse and remission alternately. It remains a great challenge to diagnose and assess disease activity during IBD due to the lack of specific markers. While traditional biomarkers from plasma and stool, such as C-reactive protein (CRP), fecal calprotectin (FC), and S100A12, can be used to measure inflammation, they are not specific to IBD and difficult to determine an effective cut-off value. There is consensus that gut microbiota is crucial for intestinal dysbiosis is closely associated with IBD etiopathology and pathogenesis. Multiple studies have documented differences in the composition of gut microbiota between patients with IBD and healthy individuals, particularly regarding microbial diversity and relative abundance of specific bacteria. Patients with IBD have higher levels of *Proteobacteria* and lower amounts of *Bacteroides, Eubacterium*, and *Faecalibacterium* than healthy individuals. This review summarizes the pros and cons of using traditional and microbiota biomarkers to assess disease severity and treatment outcomes and addresses the possibility of using microbiota-focused interventions during IBD treatment. Understanding the role of microbial biomarkers in the assessment of disease activity and treatment outcomes has the potential to change clinical practice and lead to the development of more personalized therapies.

## Introduction

Inflammatory bowel disease (IBD) including ulcerative colitis (UC) and Crohn's disease (CD), is a chronic relapse-remitting disease characterized by intestinal inflammation. The conventional approach to treatment of IBD has mainly focused on symptom relief. However, treatment that only targets symptoms can be ineffective because symptoms do not consistently reflect the presence or severity of mucosal inflammation. Instead, a “treat-to-target” management approach aims to target mucosal healing in intestinal inflammation, especially histological healing, thereby improving patient prognosis (1). Thus, treatment would be improved through frequent, objective, and regular assessments of the inflammatory process (2). Biomarkers help to correctly categorize disease severity, distinguishing between patients who have exhibited improvement or resolution of their inflammation, and those who have had a recrudescence of inflammation after medically or surgically induced remission, even prior to recurrence of clinical symptoms. Previously, endoscopy was regarded as the gold standard for gastrointestinal diseases because this tool allows for visualization of the affected tissue and the opportunity for biopsy. However, endoscopy is limited in clinical application because of its invasiveness, high cost, reliance on anesthetics, and risk of intestinal perforation (3). In recent years, many serum and feces biomarkers have been proposed to help monitor disease and assess mucosal healing to effectively diagnose residual intestinal inflammation in patients with IBD.

**Figure 1:**
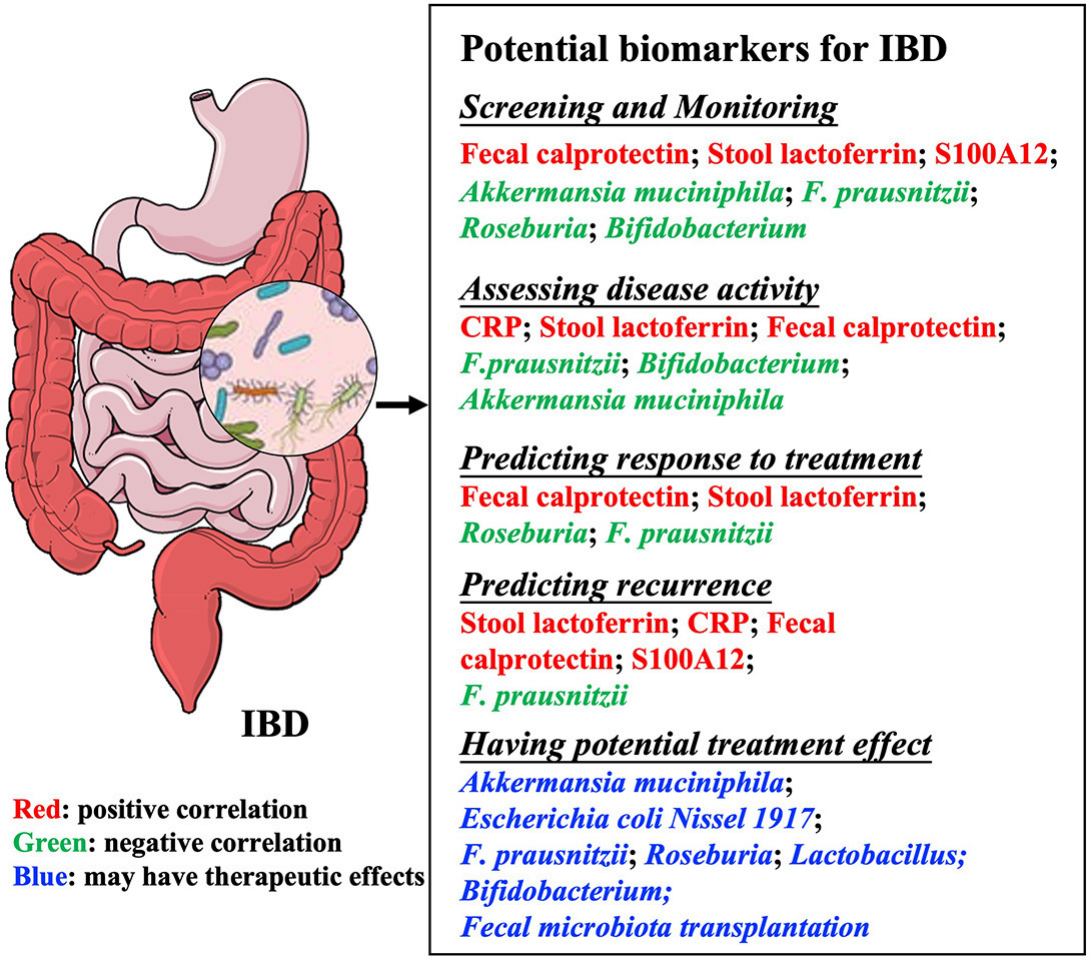
Summary of potential biomarkers in inflammatory bowel diseases. Microbial biomarkers are used for many purposes, including monitoring and evaluating disease activity, predicting recurrence or response to treatment, and treating diseases. IBD, Inflammatory bowel disease; CRP, C-reactive protein; *F. prausnitzii, Faecalibacterium prausnitzii*.

Currently, the precise etiology of IBD is still not clear, but an inappropriate and persistent inflammatory response to commensal gut microbiomes in genetically susceptible individuals are currently thought to involve the pathogenesis of IBD. Moreover, epidemiological studies have showed that several environmental exposures such as diet, smoking, hygiene, antibiotics, mode of birth (vaginal vs. cesarean section), breast feeding, infections, and stress are known common risk factors for developing IBD. And all these aforementioned risk factors are well known to cause microbial perturbations, suggesting gut microbiota play a critical role in the pathogenesis of IBD (4–7). Indeed, studies show that the intestinal microbiota in patients with IBD is distinct from that found in healthy subjects (8–10). Microbial biomarkers, which are used to assess disease activity and treatment effectiveness, are promising method to be utilized in clinical practice and optimize personalized treatment strategies. There is also interest in the potential benefits of microbiome-modulating interventions, such as probiotics, prebiotics, antibiotics, fecal microbiota transplantation (FMT), and gene manipulation in IBD treatment. This review summarizes the characteristics of classical biomarkers commonly used in assessing IBD, describes research on gut microbiota (Figure 1), and discusses the advantages and disadvantages of bacteria as biomarkers for IBD.

## Traditional Plasma and Stool Biomarkers Used to Assess IBD Activity

Laboratory tests that are fast, convenient, non-invasive, inexpensive, standardized, and repeatable, are widely used to assess disease progression. Some blood and stool biomarkers, including serum C-reactive protein (CRP), fecal calprotectin (FC), Calgranulin C (S100A12), stool lactoferrin (SL), and neutrophil-lymphocyte ratio (NLR) have been used as inflammatory indicators of IBD (Table 1). Some of these are used to distinguish IBD from irritable bowel syndrome (IBS) (24). For example, CRP is widely used in IBD screening and assessment of disease activity, clinical relapse and treatment responsiveness, serving as a predictor of surgical outcomes for a subgroup of patients with UC or CD (25, 26). In addition, baseline CRP levels, which reflect the persistence of severe disease and control of intestinal inflammation (27), can predict the response of patients with CD to anti-TNF and anti-adhesion molecule therapy (28). The high CRP group had a more severe clinical course (29) and a better response to infliximab and elevated CRP (>45 mg/L) in patients with IBD and can reliably predict the need for colon resection (30, 31). However, elevated serum CRP is not unique for intestinal inflammation, occurring in response to most systemic inflammatory disorders (14). In addition, there is considerable heterogeneity in CRP generation based on the genetics of individual patients (14). Plasma CRP is not a target for therapy, but rather a tool to monitor inflammation and guide radiology or endoscopy assessments.

**Table 1:** Commonly used biomarkers in blood and stool.

**Samples**	**Biomarkers**	**Changes in IBD**	**Advantages**	**Disadvantages**
Plasma	CRP (11, 12)	↑	1. Rapid increase in a short period. 2. Sensitive to inflammation 3. The half-life is relatively short (about 19 h).	1. Lack of specificity 2. Assays vary in their sensitivity and definitions of normal cut-off values 3. Expression of CRP is affected by many factors.
	ESR (11)	↑	Evaluation verification degree.	Low sensitivity and specificity
	NLR (13)	↑	1. Used to predict loss of response to IFX in patients with both CD and UC 2. Has utility at nearly every point in IBD management.	1. Normal range of NLR has not been precisely defined 2. Inconsistent with ESR, FC and CRP in magnitude and significance 3. Impacted by other reasons: age, sex, menopausal status, and so on.
Stool	Fecal Calprotectin (14–16)	↑	1. High negative predictive value 2. High sensitivity, helps to determine whether an endoscopy is needed, which is cost-effective 3. Good stability (~7 days at room temperature).	1. Lower specificity of FC for IBD and other inflammatory and infectious conditions 2. disease type (CD vs. UC) and disease location (colitis vs. enteritis) may be associated with distinct levels of FC.
	Calgranulin C (17–20)	↑	1. Similar diagnostic accuracy to FC 2. Only expressed in neutrophils, thus having better diagnostic performance in IBD 3. Compared with IBD, fecal lactoferrin may be a better predictor of IBS.	1. Little research on potential applications 2. Poor at predicting or monitoring responses to IBD treatment 3. Weak correlation to clinical and histological severity scores of UC patients.
	Stool lactoferrin (3, 21–23)	↑	1. Strong stability, not affected by multiple freeze-thaw cycles 2. Strong relationship between SL concentration and degree of mucosal inflammation.	1. Use is mainly limited to research 2. There is a lack of the assessment of responsiveness to disease changes.

*CRP, C-reactive protein; ESR, Erythrocyte sedimentation rate; NLR, Neutrophil–lymphocyte ratio; FC, Fecal Calprotectin; IFX, infliximab; SL, stool lactoferrin; IBS, Irritable bowel syndrome; IBD, Inflammatory bowel disease; UC, ulcerative colitis; CD, Crohn′s disease*.

Fecal calprotectin (FC) accounts for 60% of the cytoplasmic protein concentration of neutrophils and macrophages (32) and is considered the most reliable predictor of short-term recurrence and inflammatory activity in IBD (33–35). However, the cut-off value for fecal calprotectin has always been controversial and is affected by numerous factors including time of defecation (36), patient age (37), diet and lifestyle (38, 39), disease, medication usage (40), and storage conditions (36, 41). In addition, many studies have reported different cut-off values for fecal calprotectin, ranging from 100 to 250 μg/g (42, 43), such that only a moderate prediction of disease can be made for individual patients.

Calgranulin C, or S100A12, belongs to the S100 family of calcium-binding proteins. S100A12 is expressed as a cytoplasmic protein in neutrophils and has a pro-inflammatory function that includes potent chemotactic activity (44, 45). Both expression and secretion of S100A12 are increased in the serum and colon tissues from patients with IBD, and S100A12 levels in feces can be used as an indicator of mucosal healing and disease severity (15, 46). For example, S100A12 has 96% specificity for IBS vs. IBD when the threshold was set as 10 mg/kg. The odds ratio of stool S100A12 in IBD has a more accurate sensitivity, specificity, likelihood ratio, negative predictive value, and positive predictive value than FC and CRP even though studies set different thresholds (20, 47). S100A12 level can also increase in response to other diseases (48), however, the degree of daily change remains to be investigated. The lack of standardized detection methods may lead to different results (17, 49), which make it difficult to determine an accurate and effective cut-off value. In addition, there is no correlation between Crohn's Disease Activity Index (CDAI) and fecal S100A12 in patients with CD (47). Even though S100A12 has obvious advantages over FC, researchers have not yet reached a consensus, and the role of S100A12 in IBD development remains to be elucidated.

Stool lactoferrin (SL) is a multifunctional iron-binding glycoprotein. Inflammation in the intestinal lumen triggers the infiltration of pleomorphic granulocytes and neutrophils to the mucosal surface, which in turn causes a proportional increase in lactoferrin within the feces (50). There is a strong correlation between SL migration and the degree of mucosal inflammation determined by endoscopy (51). SL and FC have similar accuracy, sensitivity, and specificity (52). However, as compared with CRP and FC, SL testing has been primarily limited to research studies, likely as a result of the lower stability of lactoferrin at room temperature (53).

Of these biomarkers, fecal calprotectin (FC) correlates best with the number of active inflammation sites reported from biopsy than serum CRP and white blood cell count (15). Erythrocyte sedimentation rate (ESR), platelet count, and serum albumin level are also used to help assess IBD inflammation (54, 55), though they are not particularly accurate measures of the disease.

## The Role of Gut Microbiota in the Prediction, Diagnosis, and Treatment of IBD

The gut microbiota harbor 100 times more genes than the host genome (56). The majority of gut microbiota belong to four phyla: *Firmicutes, Proteobacteria, Bacteroidetes*, and *Actinobacteria*, with *Firmicutes* and *Bacteroidetes* predominating in healthy adults (57). Patients with UC and CD show a significant reduction in microbial diversity and alterations in some specific taxa (58) (Table 2). A prospective study of pediatric patients with CD (65) similarly concluded that gut dysbiosis is a marker for the presence and severity of inflammation. Multi-omics data supports the role of the microbiome in IBD by accurately describing the intestinal flora and its function, thus informing follow-up, diagnosis, prevention of recurrence or complications, and treatment (66). Understanding the role of microbial biomarkers in the assessment of disease activity and treatment outcomes is critical to the monitoring and treatment of IBD. A summary of recent findings relating to intestinal flora is described below.

**Table 2:** Changes in common intestinal bacteria in inflammatory bowel disease.

**Gut microbiome**	**Years**	**Country**	**Sample size**	**Age**	**Microbiology assessment**	**Abundance in IBD**	**Active UC/quiescence UC**	**Active CD/quiescence CD**
*Clostridium leptum* (59)	2013	Indian	17 HC; 20 CD; 22 UC	CD:31.2 y; UC: 38.4 y HC: 31.1 y	Real-time PCR; TTGE	↓	NS	NS
*Faecalibacterium prausnitzii* (60, 61)	2014 2011	Belgium	127 UC and 87 HC 68 CD and 139 HC	HC: 41.5 y (30–53); UC: 43 y (32–55) CD: 45 y (25–76) Unaffected relatives: 52 y (14–86) Control: 50 y (28–78)	DGGE; Real-time PCR	↓	↓	↓
*Roseburia* (60, 61)	2014 2011	Belgium	127 UC and 87 HC 68 CD and 139 HC	HC: 41.5 y (30–53); UC: 43 y (32–55) CD: 45 y (25–76) Unaffected relatives: 52 y (14–86) Control: 50 y (28–78)	DGGE; Real-time PCR	↓	↓	↓
*Bifidobacterium* (62)	2013	UK	33 UC; 18 HC	UC: 53 y (19–81); HC: 57.5 y (19–81)	Real-time PCR	↓	↓	NA
*Lactobacillus* (62)	2013	UK	33 UC; 18 HC	UC: 53 y (19–81); HC: 57.5 y (19–81)	Real-time PCR	↓	↓	NA
*Clostridiu buytricum* (62)	2013	UK	33 UC; 18 HC	UC: 53 y (19–81) HC: 57.5 y (19–81)	Real-time PCR	↓	↓	NA
*Akkermansia* (63)	2010	Australia	20 HC; 20 UC; 26 CD	HC: 53 y (22–84); UC: 48 y (24–71) CD: 38 y (19–74)	Real-time PCR	↓	↓	↓
*Ruminococcaceae* (64)	2021	China	89 UC; 33 HC	HC: 55.5 y; UC: 43 ± 3 y	16s sequencing	↓	↓	NA

*UC, patients with UC; CD, patients with CD; HC, healthy people; TTGE, temporal temperature gradient electrophoresis; DGGE, denaturing gradient gel electrophoresis; Real-time PCR, Real-time Quantitative polymerase chain reaction; NS, no significance; NA, not available; ↓, decline in quantity*.

### Intestinal Flora Can Serve as a Marker for IBD Identification

Following the rapid advance of genomics, many studies found that patients with IBD can be distinguished from patients with IBS and healthy controls based on alterations in bacterial diversity and abundance of some particular bacterial communities (67–69). The main genera reduced in fecal samples of patients with CD are *Bacteroides, Eubacterium, Faecalibacterium*, and *Ruminococcus* (70, 71). Of these, extensive research has focused on *Akkermansia muciniphila* (71) and *Faecalibacterium prausnitzii* (72). Lopez-Siles et al. (73) assessed the presence of *F. prausnitzii* and *E. coli* in 28 healthy controls, 45 patients with CD, 28 patients with UC, and 10 patients with IBS. Findings confirmed that *F prausnitzii* was a specific indicator of IBD. The abundance of *F. prausnitzii* in patients with IBD was significantly lower than in IBS patients and healthy controls (*P* < 0.001). When *F. prausnitzii* is combined with *E. coli*, it can even distinguish colonic-CD from extensive colitis (73), suggesting that a combination of multiple bacteria may be more suitable biomarkers than individual bacteria for IBD. We previously found the gut microbiota is informative enough to distinguish HC from CD or UC samples with model accuracy of 89.5 and 93.2%, respectively (68). Similarly, the model built from RISK and PRISM biopsy samples (74) achieves high prediction accuracies as well, although Western fecal samples are less informative to classify IBD from HC (68).

### Assessment of Disease Activity and Treatment Efficacy

The abundance of *Actinobacteria* and *Proteobacteria* was found to be relatively higher in patients with IBD than healthy subjects (68), while the abundance of *Firmicutes* was relatively lower. The degree of change to the flora was closely related to IBD severity. Recovery of *Clostridiales* is associated with disease remission and may be used to guide treatment. Fukuda and Fujita (72) obtained intestinal microflora from feces samples and used T-RFLP for OTU discriminant analysis, calculating standard discriminant function coefficients (Df) for each OTU. The sum of each OTU multiplied by the Df value is called the discriminant fraction (Ds). Patients in this experiment were divided into five groups, including patients with active UC (Group I), mild inflammation in the large intestine (Group IIa), no inflammation (Group IIb), consanguineous-healthy (Group III), and non-consanguineous-healthy (Group IV), The Ds values of each group decreased gradually from Group I to Group IV, suggesting that Ds may become a clinically relevant biomarker of disease activity in UC. He et al. (64) compared stool samples from patients with UC with different levels of inflammation and non-IBD controls and found lower diversity of microbiota in patients with UC, especially among those in the active stage. The abundance of *Proteobacteria* was significantly higher in patients with active UC and decreased significantly in patients in remission, while the reverse was true for *Firmicutes. Klebsiella, Enterococcus*, and *Haemophilus* were higher in patients with active UC, while *Roseburia, Lachnospira, Blautia*, and *Faecalibacterium* were higher in patients with UC who were in remission. A random-effects meta-analysis of 231 patients with CD and 392 patients with UC conducted by Prosberg et al. (75) showed that the abundance of *F. prausnitzii* was lower in patients with active CD and UC than those in remission, further suggesting that *F. prausnitzii* may be a reliable marker for assessing disease activity. Our previous study (68) also found that a relative increase in Actinobacteria and Proteobacteria (Enterobacteriaceae) and decrease in Firmicutes (Clostridiales) were strongly correlated with IBD severity by analyzing fecal microbiota in both UC and CD with mild, moderate or severe activity.

Anti-TNF treatment of IBD can trigger and maintain remission, improve quality of life, and alter the course of the disease (76, 77). However, nearly one-third of patients do not respond to anti-TNF treatment. Research indicates that changes in gut microbiota can reliably identify patients with CD responsiveness to TNF therapy, which can improve clinical management, reduce morbidity, and improve symptoms in these patients. Sanchis-Artero et al. (77) divided 27 CD patients initiating anti-TNF treatment into responders and non-responders to evaluate *F. prausnitzii/Escherichia coli* and *F. prausnitzii/C. coccoides* ratios before and after 6 months of treatment. Results suggest that the *F. prausnitzii/Escherichia coli* ratio can serve as a reliable early biomarker for identifying anti-TNF responsiveness in patients with CD. Kugathasan et al. (78) showed *Veillonella* was implicated in penetrating complications and *Ruminococcus* in stricturing CD in a prospective initial cohort study in children with CD. In our previous study, we also conclude that certain microbes, mainly Clostridiales, predicted the infliximab treatment effectiveness with 86.5% accuracy alone and 93.8% when combined with calprotectin levels and Crohn's disease activity index (CDAI) in a small CD cohort (68).

### Prediction of Clinical Relapse

Approximately 50–75% of patients with CD need a bowel resection during the disease course (79), and 50% of patients have recurrent symptoms after surgery (80). To evaluate the risk of postoperative recurrence, ileal colonoscopy is currently recommended within 1 year after surgery to evaluate endoscopic recurrence using the Rutgeerts score. Currently, colonoscopy is still the gold standard for diagnosis and disease follow-up (81, 82), but a recent study suggests intestinal flora as a non-invasive marker. Pascal et al. (83) evaluated the predictive value of microbiota for postoperative recurrence using Kruskal-Wallis analysis of stool samples collected preoperatively from 54 patients and comparing them with the Rutgeerts score at 6 months after surgery. *Streptococcus* levels were higher in patients with postoperative recurrence, suggesting that the presence of these bacteria in preoperative stool samples may be a predictive marker of future recurrence. A reduction in *F prausnitzii* levels is related to a higher risk of recurrence in ileal CD. Soko et al. (84) analyzed the mucosal microflora of patients with CD at the time of surgical resection and 6 months post-surgery and found that a decrease in *F. prausnitzii* was related to endoscopic recurrence after 6 months. *F. prausnitzii* was positively correlated with the duration between flare-ups, with higher levels associated with longer remission times. Sokol et al. (85) compared the ileal microbiota of 201 patients in ileostomies at the time of resection and over the following year using 16S rRNA sequencing. Results showed that the abundance of ileal microbiota, including *Gammaproteobacteria, Corynebacterium*, and *Ruminococcus gnavus*, during resection, was significantly correlated with the risk of endoscopic recurrence. In addition, mounting evidence suggests that baseline gut microbiota may influence response to medication (86). Radhakrishnan et al. (87) conducted a systemic review by analyzing 19 articles that related to the association between anti-IBD drug treatment and gut microbiota. In this study, they concluded that compared to the IBD non-responders, those responders to enteral nutrition (EEN), infliximab, ustekinumab, and vedolizumab treatment have an increase in baseline microbial alpha diversity. Moreover, higher baseline *Faecalibacterium* levels can also predict responsiveness to ustekinumab and infliximab treatment.

### Use of Intestinal Flora to Treat IBD

During the last years, the therapeutic targets for IBD are focusing on “target” to treat that target the stage of mucosal healing in intestinal inflammation especially histological healing (88, 89). A significant proportion of patients with IBD are resistant to treatment using standard drugs. Even using optimal doses of 5-aminosalicylic acids (5-ASA) and/or azathioprine, annual recurrence rates can be as high as 25–40% (90). Introducing probiotics and prebiotics into the diet can help to restore the natural flora, prevent pathogenic bacteria infection by producing antimicrobial peptides and promote intestinal health by stimulating the growth and activity of beneficial bacteria through prebiotic fermentation. *Bifidobacteria, lactobacillus*, VSL#3, and butyric acid-producing bacteria have been used in clinical treatment. Probiotics effectively regulate the imbalance of intestinal flora, improve the microecological environment, enhance the intestinal mucosal barrier function, modulate the local and systemic immune responses, and provide new options for the treatment of diseases like IBD (91, 92).

Furthermore, researchers were also trying to address the potential of probiotic cocktails for IBD. For example, VSL3#, a mixture of eight probiotic cocktails, could upregulate the antagonists of NF-κB inflammatory pathway (93). Oral administration of 17 strains of bacteria to adult mice could attenuate colitis (94). Toumi et al. (95) reported that administration for a week of Ultrabiotique (*Lactobacillus acidophilus, Bifidobacterium lactis, L. plantarum, and Bifidobacterium breve*) can augment the production of intestinal mucus and goblet cells per crypt in mice, suggesting that probiotics may be a promising therapeutic intervention in situations that require immediate mucosal healing. Moreover, a four probiotic mixture containing *L. plantarum, L. acidophilus, Enterococcus faecium*, and *L. rhamnosus* can also exert positive effects on wound healing by increasing the wound healing rate of epithelial cells *in vitro* and enhancing the integrity of their tight junctions' formation (96). Dharmani et al. (97) reported that a mix of eight different probiotic bacteria promoted ulcer healing in rats through the induction of vascular endothelial growth factor (VEGF). These studies suggest probiotics have key roles in tissue repair or mucosal healing.

Currently, Fecal Microbiota Transplantation (FMT) is a novel UC treatment for intestinal microbiome disorders (98). Transplantation of functional bacteria from the stools of healthy people into the gastrointestinal tract of patients, and the reconstruction of new gut microbiota is an effective treatment for both intestinal and extra-intestinal disorders. FMT reduces intestinal permeability and disease severity by enhancing the production of short-chain fatty acids (SCFAs), particularly butyric acid, which helps maintain intestinal epithelial barrier integrity (99). FMT can also restore immune dysregulation by inhibiting Th1 differentiation, T cell activity, leukocyte adhesion, and the production of inflammatory factors (100, 101). Several multicenter, randomized, double-blind, placebo-controlled trials indicate that FMT induces remission in patients with active UC (100–103). In these studies, patients with active UC were divided into FMT and placebo groups. Remission rates were significantly higher in the FMT group than in the placebo group, with no difference in the incidence of adverse events. Treatment was most successful for newly diagnosed patients with UC, possibly because the microbiome was not as severely impacted by the disease. Although studies show that the intestinal flora of the host and donor are very similar approximately 2–4 weeks after FMT (104), it remains uncertain whether there are potential sequelae. Several questions still need to be addressed to optimize FMT treatment, including whether or not glucocorticoids should be discontinued during treatment, how time and intensity of intervention impact therapeutic effect, what inclusion criteria should be considered for the donor, whether single or multiple donors should be used for FMT, and whether aerobic or anaerobic treatment should be used for fecal treatment. Larger, long-term, multicenter studies with sufficient sample sizes and detailed microbiome analyses of both patients and donors will be needed to help inform UC treatment.

## Discussion

Diagnosis and management of IBD have evolved substantially in recent decades. Observational studies evaluating IBD disease outcomes have shown a reduction in surgery incidence, likely resulting from the development of novel therapeutics (105). While fecal calprotectin and other non-invasive markers of disease activity have allowed physicians to define disease activity more objectively, individual differences in response to specific drugs can affect treatment efficacy and/or toxicity. For example, 60% of patients with UC were shown to respond to mesalazine or 5-ASA treatment, suggesting that 40% of patients may not experience any benefit or may even have adverse drug effects (106). Primary non-responders have also been observed in 20–30% of patients with anti-TNFα treatment, vedolizumab, and/or ustekinumab treatment. This high rate of non-responsiveness may in part be explained by incomplete patient assessment preceding biological therapy. Therefore, it would be of great value to accurately predict responsiveness before therapy, but clinical predictors have proven insufficient and targeted markers are still lacking. For example, Hyams et al. (107) recruited pediatric patients aged 4–17 years with newly diagnosed UC from 29 centers in the USA and Canada. Findings supported the utility of assessing initial clinical activity and 4-week treatment responsiveness to mesalazine, showing 52-week glucocorticoid-free remission in children newly diagnosed with UC. Understanding individual clinical and biological disease features informs treatment decisions for UC. However, because up to one-third of patients without evidence of mucosal inflammation still present with gastrointestinal symptoms (108), there is clearly a need for more accurate markers to help guide clinical diagnosis.

Microbial biomarkers may also be useful to evaluate disease severity in individuals (64), helping to predict and monitor response to intervention. Endoscopic recurrence is linked to alterations of mucosa-associated microbiota in the ileal (109). Gut microbiota may aid clinicians in defining patient risk of postoperative relapse. Early prophylactic therapy can be initiated based on intestinal microbiome stratification (91). Current insights may also help in the design of microbiota modulation strategies to improve IBD outcomes (100). Studies described here present promising evidence that the intestinal bacteria may aid disease monitoring and enhance treatment efficacy for IBD like chemotherapy and immune checkpoint blockade improve cancer treatment (58, 66). Universally, higher baseline richness (without significant differences in baseline disease activity) and microbial diversity are linked to better outcomes, and less diverse gut microbiome is associated with more severe disease status.

In IBD, gut microbiota dysbiosis is associated with disease phenotypes and may be a causative or synergistic factor in prolonged or chronic inflammation. Thus, microbial treatment by restoring intestinal flora in patients with IBD has been attempted by an increasing number of scholars (110). For example, over the past decades, the frequency of clinical probiotic use has gradually increased, however, its efficacy has remained controversial. Several randomized clinical trials have shown that *E. coli Nissle* 1917 (111), VSL#3 (112), *Bifidobacterium* (113, 114) and other probiotics are effective in inducing remission or maintaining remission in IBD. Several studies have suggested that a decrease in the abundance of butyrate-producing bacteria is one of the key hallmarks of gut dysbiosis seen in IBD (60, 115, 116). Ideally, future research will focus on developing microbial-target treatments that restore gut microbial community homeostasis currently decreasing in IBD. There are promising outcomes for FMT in IBD therapy, with most patients achieving medication-free symptomatic control, and/or clinical remission (117).

The gut microbiome includes many potential biomarkers associated with disease activity and treatment outcomes in patients with IBD. Current insights may also help to design microbiota modulation strategies needed to improve IBD outcomes. Despite great advances in the microbiome field over the last decade, however, there are still a great number of issues that will need to be addressed before these findings are translated into effective therapeutic applications. While fecal flow research may be limited in its ability to detect microorganisms associated with the mucosal layer and thus directly involved in the initiation and continuation of disease, the application of this marker is far less invasive and allows for multiple and reproducible material collection, improving the monitoring of disease progression. First, most previous data were from cross-sectional rather than prospective longitudinal cohorts. Studies also differed in the information collection from IBD patients as many relevant important confounding information including diet, body mass index (BMI), antibiotic use, inflammation severity, and treatment interventions, etc. are determined to influence the gut microbiota (118). Second, the established studies mainly focus on the composition, especially the comparison of relative microbial abundances rather than the function of gut microbiota, which may be affected by total microbial counts and changes in the abundance of other species. Further, one major challenge with existing data is represented by the wide variability in the outcome definition. For example, the follow-up length, the number of outcomes assessed, and criteria met on the risk of bias assessment differed among the studies. Finally, an important limitation of existing studies is the lack of reproducibility of the microbiome in IBD. Most prediction studies did not include independent external validation cohorts. In addition, microbiome studies only focused on bacteria and did not explore other microorganisms such ad fungi and viruses, as well as how they interact with each other (10). Furthermore, studies have also varied in the depth of microbial sequencing, from PCR–based methods to 16s rRNA profiling to metagenomics sequencing and strain-level analysis. In short, this is still an open field and active research area for the investigation of gut microbiota in patients with IBD.

## Author Contributions

XG, CH, and JX are involved in the designing and drafting of the manuscript. HX, LL, HZ, JW, WH, and WP are responsible for searching and screening papers, and manuscript revision. YN and YC interpretation of the data and revision of the article. YouZ and YonZ concept and design of the study, interpretation of the data, and revision of the article. All authors contributed to the paper and approved the submitted version.

## Funding

This work was supported by the grants from National Natural Science Foundation of China (No. NSFC 81700487), Natural Science Foundation of Guangdong Province (No. 2020A1515011000), Guangzhou Planned Project of Science and Technology (Nos. 202002030293 and 202002020012), Innovative Clinical Technique of Guangzhou (No. 2019GX05), and Projection of building a state-level synthesize hospital demonstrating traditional Chinese medicine and west medicine (No. 3012000000261).

## Conflict of Interest

The authors declare that the research was conducted in the absence of any commercial or financial relationships that could be construed as a potential conflict of interest.

## Publisher's Note

All claims expressed in this article are solely those of the authors and do not necessarily represent those of their affiliated organizations, or those of the publisher, the editors and the reviewers. Any product that may be evaluated in this article, or claim that may be made by its manufacturer, is not guaranteed or endorsed by the publisher.
